# *KIBRA* gene polymorphism has no association with verbal or visual episodic memory performance

**DOI:** 10.3389/fnagi.2014.00270

**Published:** 2014-10-08

**Authors:** Katherine H. Franks, Mathew J. Summers, James C. Vickers

**Affiliations:** Wicking Dementia Research and Education Centre, Faculty of Health, University of TasmaniaHobart, TAS, Australia

**Keywords:** *KIBRA*, *WWC1*, episodic memory, aging, single-nucleotide polymorphism

## Abstract

Inter-individual variability in memory performance has been suggested to result, in part, from genetic differences in the coding of proteins involved in long-term potentiation (LTP). The present study examined the effect of a single-nucleotide polymorphism (SNP) in the *KIBRA* gene (rs17070145) on episodic memory performance, using multiple measures of verbal and visual episodic memory. A total of 256 female and 130 male healthy, older adults (mean age = 60.86 years) were recruited from the Tasmanian Healthy Brain Project (THBP), undergoing both neuropsychological and genetic testing. The current study showed no significant effect of the *KIBRA* polymorphism on performance on the Rey Auditory Verbal Learning Task, Logical Memory test, Paired Associates Learning test or Rey Complex Figure Task. The results suggest there is little to no functional significance of *KIBRA* genotype on episodic memory performance, regardless of modality.

## Introduction

The term “memory” encapsulates different systems that can be differentiated by the type of information processed. Declarative memory, one of two major distinctions of memory, involves the conscious recollection about facts (semantic memory) and events (episodic memory; Squire, 2004). Decline in memory performance, particularly episodic memory, is a major characteristic of dementia, particularly in early-middle stages. However, it is also well established that there is normal aging-associated decline of episodic memory in the absence of dementia (Mormino et al., 2009). Therefore, with aging populations, it is not only important to investigate strategies to delay dementia onset, but also to increase resistance to aging-related cognitive decline. As the heritability estimate of memory ability is approximately 50% (McClearn et al., 1997), genetic components have been investigated to determine whether they have either a protective effect on, or predict a faster decline of, normal aging-associated cognitive decline. The association of genes such as brain-derived neurotrophic factor (*BDNF*) and apolipoprotein-E (*ApoE*) with cognitive decline and late-onset Alzheimer’s disease (AD) has been investigated (e.g., Corder et al., 1993; Slooter et al., 1998; Hariri et al., 2003; Cosentino et al., 2008). Such insight assists in informing the development of strategies to increase resistance to memory decline in older adults.

It has been proposed that the *KIBRA* gene (also known as *WWC1*) may also play a role in human memory (Papassotiropoulos et al., 2006), potentially accounting for some of the individual variability in memory decline. The *KIBRA* single-nucleotide polymorphism (SNP) (rs17070145) was originally identified in a genome-wide association study (GWAS), as it met criteria of being associated with memory performance (Papassotiropoulos et al., 2006). Follow-up studies demonstrated that the C → T substitution within the ninth intron of the *KIBRA* gene was significantly associated with greater episodic memory performance (Papassotiropoulos et al., 2006). Further, there was no significant relationship between genotype and performance on attention, concentration and working memory tasks, suggesting that *KIBRA* has a specific association with episodic memory. Additionally, this study also demonstrated that T-allele carriers had significantly better visual episodic memory performance than non-carriers, suggesting the effect of *KIBRA* genotype is independent of stimulus modality.

The *KIBRA* gene has been shown to encode KIBRA protein, which is expressed in brain regions related to memory, including the hippocampus and temporal lobe in humans (Papassotiropoulos et al., 2006). Animal studies provide preliminary evidence that the gene and associated protein are implicated in cellular processes underlying memory and, thus, are related to memory performance. This is illustrated by adult *KIBRA* knockout mice demonstrating significant deficits in learning and memory, specifically in long-term potentiation (LTP) and long-term depression in the hippocampus (Makuch et al., 2011). Long-term potentiation has been proposed to underlie memory formation, involving the activation of proteins, gene expression, protein synthesis and the development of synaptic connections (Lynch, 2004). There is evidence to suggest that *KIBRA* may be involved in LTP, as KIBRA protein interacts with proteins that play a role in altering synaptic plasticity for memory formation, such as protein kinase M zeta, dendrin and synaptopodin (Kremerskothen et al., 2003; Schneider et al., 2010).

Multiple investigations have subsequently investigated the association between *KIBRA* T-allele and superior verbal episodic memory performance. While many studies provide further support for this association (Almeida et al., 2008; Schaper et al., 2008; Bates et al., 2009; Preuschhof et al., 2010; Kauppi et al., 2011; Wang et al., 2013), some report no association between *KIBRA* genotype and verbal episodic memory performance (Need et al., 2008; Burgess et al., 2011; Wersching et al., 2011; Laukka et al., 2013; see Schneider et al., 2010 for a review). In addition to this, the *KIBRA* T-allele has also been reported to have a deleterious effect, whereby T-allele carriers have worse performance on verbal episodic memory tests (Nacmias et al., 2008). Despite this, the majority of results from studies investigating the effect of *KIBRA* genotype are consistent with the original findings. Further, studies utilizing relatively small samples (e.g., Schaper et al., 2008) still demonstrate an effect of *KIBRA* on episodic memory performance (Rey Auditory Learning Verbal Learning Test, RAVLT), suggesting that the effect of *KIBRA* on episodic memory may be of a sufficient magnitude to be detected within smaller samples.

The aim of the present study was to clarify the effect of *KIBRA* polymorphism on episodic memory performance of healthy aging individuals, using multiple measures of both verbal and visual episodic memory, and by examining immediate and delayed recall.

## Materials and methods

### Participants

The sample in the present study consisted of 386 participants who were drawn from the Tasmanian Healthy Brain Project (THBP), a world-first, large-scale longitudinal study investigating the protective effect of later-life education on age-related cognitive decline in people aged between 50 and 79 years (Summers et al., 2013). The sample consisted of 256 females and 130 males, with a combined mean age of 60.86 (*SD* = 6.79). Participants were excluded from the THBP if they had a history of medical, psychological or psychiatric conditions independently associated with impairments to cognitive function (see Summers et al., 2013). All participants provided informed consent prior to commencing the study and ethics approval was obtained from the Tasmanian Social Sciences Human Research Ethic Committee (H11070).

### Genotyping

Oragene DNA Self-Collection Kits (DNA Genotex, Ottawa, Canada) were used to collect saliva samples for DNA extraction, which was carried out using standard methods. *KIBRA* polymorphism was determined using restriction polymerase chain reaction-fragment length polymorphism (PCR-RFLP) analysis. Similarly to Wersching et al. (2011), two primers were used to select a 198 basepair (bp) fragment containing the polymorphic site rs17070145 (forward: atcctcttgaggcttcactgg, backward: actttcaacacaatgaacaagg). The total volume of amplification reactions was 12 µl, consisting of 1 µl of DNA, 0.6 µl of each primer, 6 µl of MyTaq DNA polymerase and 3.8 µl of DEPC treated water. Following the PCR program, the products were then digested in 5 U of *MseI* restriction enzyme for 16 h at 37°C. As there is no *MseI* site in the C-allele, but this is present in the T-allele, the PCR product was cut into two fragments of 128 and 70 bp in length only when the T-allele was present. Therefore, a single band at 198 bp indicated a CC genotype, whereas a band at both 128 bp and 70 bp indicated a TT genotype. Bands at all three bp locations indicated a CT genotype. Polymerase chain reaction products were then viewed using 3.5% agarose gels stained with Sybr-safe to allow visualization of DNA. All samples were genotyped twice to ensure accuracy, with 87.3% of samples in concordance. In the instance of conflicting results, the triplicate result was taken.

### Neuropsychological testing

As part of the THBP, participants underwent annual cognitive assessment requiring participants to complete a standardized battery of neuropsychological measures selected to assess core cognitive function. A qualified examiner administrated the tests in an individual assessment session as part of standard protocol of the THBP (described by Summers et al., 2013). The cognitive measures analyzed in the current study formed a part of the larger cognitive battery. Verbal episodic memory performance was assessed using the RAVLT and the Logical Memory Test (LM; a subtest from the Wechsler Memory Scale—Third Edition; WMS-III). Visual episodic memory performance was assessed using the Rey Complex Figure Test (RCFT) and the Paired Associates Learning Task (PAL; a test from the Cambridge Neuropsychological Test Assessment Battery, CANTAB).

The RAVLT (Strauss et al., 2006) is a serial word list-learning task in which 15 words are presented across five trials (list A), with recall assessed after each trial. Recall for an interference list of 15 words is then assessed (list B), followed by delayed recall of the initial list of 15 words (list A recall). The LM test (Strauss et al., 2006) assesses a participant’s capacity to recall two prose passages immediately after presentation and again following a 30-min delay.

The RCFT (Strauss et al., 2006) assesses participants’ capacity to recall a complex geometric design. The participant was shown a standardized design, which they were asked to reproduce as best as possible while it remained visible to them. Following this, both the stimulus figure and the participant’s copy were removed from view. After a delay of 20–30 min, the participant redrew the design from memory, without prior warning that they would be required to do so. The design was scored based on the accurate placement and reproduction of 18 specific design elements, with higher scores indicating greater performance.

The PAL test (Cambridge Cognition Ltd., 2004) was conducted on a touch-screen computer that displayed six boxes opening in a random order. One or more boxes contained a pattern, which at the end of all the boxes opening, were displayed individually in the middle of the screen. Participants then had to touch the box where the pattern was originally located. If an error was made, the patterns were re-presented to remind the participant of the location. Throughout the test, the difficulty increased by increasing the number of patterns presented.

These memory tests have established high levels of reliability and validity and have been used extensively to assess episodic memory in age-related cognitive decline, mild cognitive impairment and dementia (Strauss et al., 2006).

### Analysis

Differences in episodic memory performance were compared across the three *KIBRA* genotype groups: homozygous CC, homozygous TT and heterozygous CT. The three groups were dummy coded prior to analyses (0 = CC, 1 = CT, 2 = TT). The CT and TT genotypes were also combined for further analysis with respect to the CC group, to examine the effect of inheritance of any T variant.

Raw scores from the four tests of episodic memory were used for the analyses, as the correlations between age and raw scores were small (*r* = <0.3), suggesting that z-score transformations were not required. One-way ANOVAs were used to compare the mean scores on the RAVLT, LM test, PAL test and RCFT across the three groups. Assumptions of homogeneity of variance were assessed using Levene’s test and were met unless explicitly stated. Further, two 3 (Genotype: CC, CT, TT) × 5 (Trial: 1, 2, 3, 4, 5) mixed ANOVAs were conducted to assess the effect that genotype has on learning in order to determine whether an earlier stage of memory may be susceptible to genotype effects. Violations of the assumption of sphericity were adjusted using Greenhouse-Geisser corrections.

In addition, linear hierarchical regression analyses were conducted to determine whether the demographic variables of age, intelligence, previous education and gender mask the effect of genotype on episodic memory performance. This allowed the variance of such variables to be partialled out, demonstrating the underlying relationship between genotype and episodic memory performance.

Analyses were performed using IBM SPSS version 21.0.

## Results

Genetic analysis demonstrated that 176 participants were genotyped as CC carriers, 180 as CT carriers and 30 as TT carriers of the *KIBRA* gene. The observed genotype distribution was not significantly different from the frequency distribution previously observed in an Australian sample (Almeida et al., 2008; χ^2^ (2, *N* = 386) = 2.04, *p* = 0.361), suggesting that the sample used in the present study is in Hardy-Weinburg equilibrium. There were no significant differences between the three genotype groups in regards to age, sex, years of previous education, premorbid intellectual capacity (Wechsler Test of Adult Reading) or current intellectual capacity (Wechsler Adult Intelligence Scale-III-SF1; see Table 1).

**Table 1:** **Difference between *KIBRA* genotype groups on demographic variables**.

	CC (*n* = 176)	CT (*n* = 180)	TT (*n* = 30)
**Measure**	*M* (*SD*)	*M* (*SD*)	*M* (*SD*)	*p*
**Age–years**	60.27 (6.62)	61.34 (7.14)	61.37 (5.44)	0.302
**Sex ratio (males:females)**	59:117	66:114	5:25	0.100
**Prior years of education**	14.11 (2.84)	13.81 (2.58)	13.78 (2.95)	0.548
**WTAR estimated FSIQ**	112.84 (5.15)	111.87 (10.10)	113.17 (6.24)	0.846
**WAIS-III-SF1 FSIQ**	120.15 (13.37)	119.34 (12.95)	119.77 (12.65)	0.445

*Note: WTAR = Wechsler Test of Adult Reading, FSIQ = full scale intelligence quotient, WAIS-III-SF1 = Wechsler Adult Intelligence Scale 3rd edition Short Form 1*.

### Verbal episodic memory performance

Performance on the RAVLT and the LM test was assessed using three measures from each test. Rey Auditory Learning Verbal Learning Test performance was assessed by the total number of words recalled from all trials, mean number of words recalled from list A and mean number of words recognized from list A. Performance on the LM test was assessed by scores of immediate recall from the first story, delayed recall from the second story and mean percent retention from the second story. No significant differences between *KIBRA* genotype groups on any measure of verbal episodic memory performance were present (*p* > 0.05; see Table 2). There was no statistical difference between T and non-T carriers in performance.

**Table 2:** **Verbal episodic memory performance across *KIBRA* genotype**.

	CC (*n* = 176)	CT (*n* = 180)	TT (*n* = 30)
**Measure**	*M* (*SD*)	*M* (*SD*)	*M* (*SD*)	*p*
**RAVLT Total recall**	53.06 (9.09)	52.18 (9.00)	54.67 (7.22)	0.315
**RAVLT List A recall**	11.15 (2.72)	10.90 (2.80)	11.83 (2.14)	0.201
**RAVLT List A recognition**	13.49 (1.80)	13.28 (1.94)	13.67 (1.35)	0.405
**LMI immediate recall**	48.18 (8.28)	48.21 (7.96)	49.77 (6.23)	0.585
**LMII delayed recall**	30.05 (6.53)	30.06 (6.21)	31.03 (5.95)	0.719
**LMII percent retention**	87.45 (11.23)	86.85 (10.99)	87.94 (8.93)	0.819

*Note: RAVLT = Rey Auditory Verbal Learning Task, LM = Logical Memory Test*.

**Figure 1:**
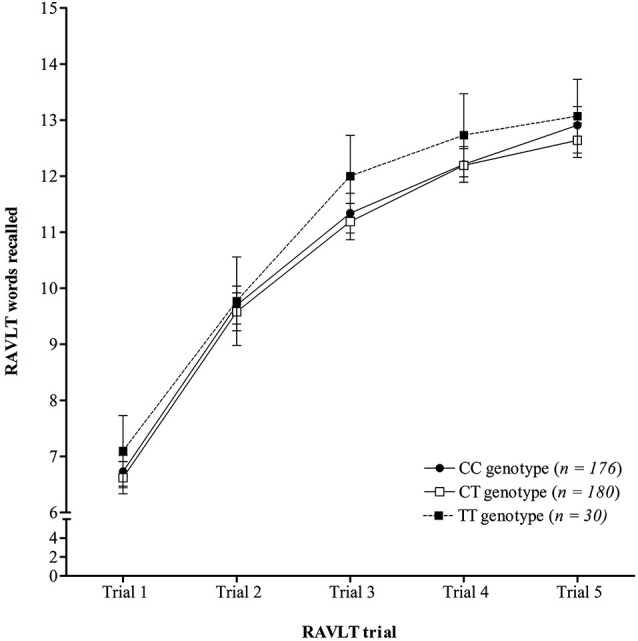
**Mean number of words recalled on each trial of the RAVLT across *KIBRA* genotype, showing no significant interaction**. Error bars display 95% confidence intervals.

Analysis of learning over trials on the RAVLT (Figure 1) demonstrated there was no significant interaction between trial number and *KIBRA* genotype on mean number of words recalled (*F*_(6.60,1263.45)_ = 0.62, *p* = 0.732, *η*_p_^2^ = 0.003), following a Greenhouse-Geisser correction (*ɛ* = 0.83), see Figure 1. The ANOVA did show a significant main effect of trial (*F*_(3.30,1263.45)_ = 680.24, *p* < 0.001, *η*_p_^2^ = 0.640), following a Greenhouse-Geisser correction, but the main effect of genotype was not significant (*F*_(2,383)_ = 1.01, *p* = 0.360, *η*_p_^2^ = 0.005).

### Visual episodic memory performance

Performance on the PAL test and the RCFT was assessed using three and two measures of memory performance, respectively. Paired Associates Learning Task test performance was assessed using mean adjusted total errors, mean trials to success and mean total errors when six shapes were presented. Performance on the RCFT was assessed using mean recall and percent retention (the ratio of what the participant recalled relative to what they copied). No significant differences between *KIBRA* genotype groups existed on any measure of visual episodic memory performance (*p* > 0.05; see Table 3). In addition, there was no statistical difference between T and non-T carriers in performance.

**Table 3:** **Visual episodic memory performance across *KIBRA* genotype**.

	CC (*n* = 176)	CT (*n* = 180)	TT (*n* = 30)
**Measure**	*M* (*SD*)	*M* (*SD*)	*M* (*SD*)	*p*
**PAL Total errors adjusted**	18.49 (19.86)	18.63 (19.43)	18.03 (22.23)	0.988
**PAL Mean trials to success**	1.72 (0.53)	1.72 (0.49)	1.65 (0.42)	0.761
**PAL Total errors 6 shapes**	6.30 (7.60)	5.69 (7.00)	5.57 (9.41)	0.751
**RCFT Recall**	21.87 (6.03)	22.11 (6.38)	21.70 (5.53)	0.908
**RCFT Percent retention**	61.79 (16.58)	62.26 (17.34)	61.40 (15.00)	0.948

*Note: PAL = Paired Associates Learning Test, RCFT = Rey Complex Figure Task*.

**Figure 2:**
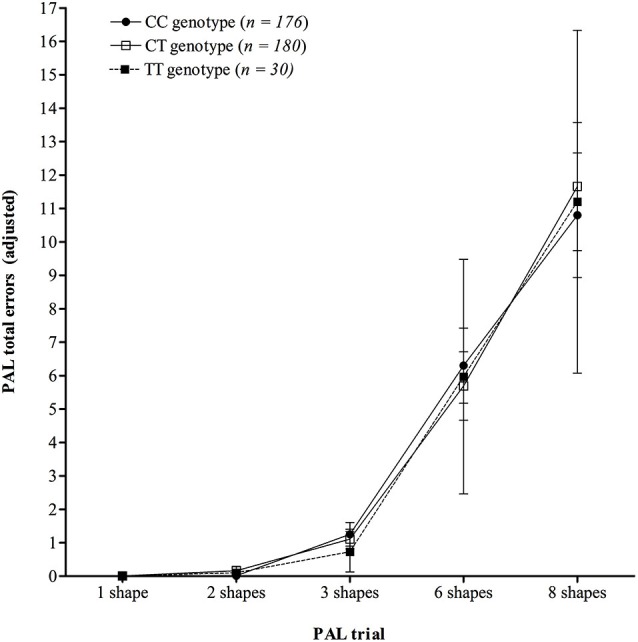
**Mean number of total errors made across trials on the PAL test, showing no significant interaction between *KIBRA* genotype**. Error bars display 95% confidence intervals.

Analysis of learning over trials on the PAL test (Figure 2) demonstrated that there was no significant interaction between trial number and *KIBRA* genotype on mean number of errors made (*F*_(2.88,551.44)_ = 0.35, *p* = 0.784, *η*_p_^2^ = 0.002), following a Greenhouse-Geisser correction (*ɛ* = 0.36), see Figure 2. The ANOVA did show a significant main effect of trial (*F*_(1.44,551.44)_ = 128.24, *p* < 0.001, *η*_p_^2^ = 0.251), following a Greenhouse-Geisser correction, but the main effect of genotype was not significant (*F*_(2,383)_ = 0.01, *p* = 0.988, *η*_p_^2^ < 0.001).

### Hierarchical linear regression

Hierarchical linear regression analyses were used to determine the underlying relationship between *KIBRA* genotype and episodic memory performance after the variance attributed by known demographic variables of age, gender, prior education, WTAR estimated IQ, and WAIS-III-SF1 FSIQ had been partialled out. The analyses demonstrated that *KIBRA* genotype, entered in the second step, resulted in a non-significant change in the amount of variance explained above that of the demographic variables for all measures of both verbal and visual episodic memory performance (*p* > 0.05).

## Discussion

This study sought to examine the effect of *KIBRA* polymorphism, particularly specific genotypes, on verbal and visual episodic memory performance of healthy aging individuals. The CC genotype of the *KIBRA* was not associated with significantly lower performance on the RAVLT, the LM test, the PAL test and the RCFT compared to those with CT or TT genotypes. The results provide no evidence of a gene-dose relationship present for the T-allele, as there was no advantage to heterozygous T-allele carriers (CT genotype) over non T-allele carriers (CC genotype). These findings suggest that *KIBRA* polymorphism has little to no influence on either verbal or visual episodic memory performance. The present study incorporated multiple measures of both verbal and visual episodic memory to ensure reliable assessment and to examine different components of memory, such as immediate and delayed recall. As there was no effect of *KIBRA* polymorphism on either delayed or immediate recall across all tests of episodic memory, this suggests that the individual polymorphism effect does not selectively influence only one component of memory. Further, analyses of the PAL test and the RCFT learning curves suggest that *KIBRA* polymorphism does not have an effect on the rate of acquisition of information (learning), indicating that *KIBRA* polymorphism does not affect an earlier stage of memory. In addition, the linear hierarchical regression analyses demonstrated no significant contribution of *KIBRA* polymorphism to the variance explained in verbal or visual episodic memory performance, despite suggestions that the effect of *KIBRA* polymorphism might be masked by other factors (Burgess et al., 2011).

The finding that *KIBRA* polymorphism does not affect verbal episodic memory performance clearly contradicts the findings of the Papassotiropoulos et al. (2006) study, which demonstrated differential performance between T-allele and non T-allele carriers. Differences between the genotype groups on delayed, but not immediate recall, was also not supported by the present study. This may be a result of delayed free recall being dependent on a large-scale memory network, rather than one specific area related to memory (Dickerson and Eichenbaum, 2010). Therefore, the delayed free recall test may be relatively non-specific and involve other brain areas where the KIBRA protein is not as highly expressed and other proteins may exert some influence on episodic memory performance.

The finding that the *KIBRA* polymorphism does not affect visual episodic memory performance also contrasts with the results from the original study showing a significant difference between T-allele carriers and non-carriers (Papassotiropoulos et al., 2006). However, it does provide support for the results of Vassos et al. (2010) and Yasuda et al. (2010), which both demonstrated that *KIBRA* polymorphism does not significantly affect visual episodic memory performance. A standardized test of visual episodic memory was not used in the Papassotiropoulos et al. (2006) study and was the only study reporting an effect on visual episodic memory, perhaps as the test may have assessed more than just visual episodic memory ability.

It has been suggested that discrepancies in, or failure to replicate findings in genetic research may be due to false positives originally being reported as true (Kathiresan et al., 2004). For example, although Schaper et al. (2008) supported the original findings of Papassotiropoulos et al. (2006), the small to medium Cohen’s *d* effect sizes reported (0.48 and 0.74) suggest approximately 57% to 67% overlap between the groups on scores of episodic memory performance (Zakzanis, 2001), which may not be functionally significant. Further, the Papassotiropoulos et al. (2006) study reported the significant difference between groups on delayed memory as only a difference of roughly 1.2 words, indicating a small effect size on performance. This is further supported by a meta-analysis demonstrating that the *KIBRA* polymorphism potentially explains only 0.5% of the variance in episodic memory performance (Milnik et al., 2012). Hence, very large sample sizes may be required to uncover such minor effect size linkages between the *KIBRA* polymorphism and memory performance. In this regard, it may be possible that *KIBRA* gene variations may combine with other brain gene polymorphisms (e.g., *CLSTN2*, Preuschhof et al., 2010, but not seen in Sédille-Mostafaie et al., 2012; Laukka et al., 2013) to influence memory performance, albeit effect sizes are likely to remain small.

With respect to potential limitations of the current study, as the THBP is a longitudinal study that invites older individuals to attend university to increase their education, this may introduce some bias in the recruitment of participants. For example, only motivated individuals or those who completed secondary school or have previous university experience may be willing to participate, which would explain the above average levels of intelligence and premorbid IQ seen in the current sample. Consequently, this may impact on any generalizability of findings to different populations. However, the THBP sample features did not substantially differ from the previous *KIBRA* studies that reported prior years of education and intelligence scores (see Table 4).

**Table 4:** **Comparison of the sample features in the present study to other studies**.

Study	Years of prior education	Full scale IQ
Schaper et al. (2008)	14.1–15.1	122.4–123.7 (VIQ)
Preuschhof et al. (2010)	12.4–13.3	N/A
Yasuda et al. (2010)	15.5–15.7	108.8
Current study	13.78–14.11	119.75

*Note: N/A = not available, VIQ = verbal intelligence quotient*.

The present study has extended the findings of previous research investigating the *KIBRA* gene, particularly through the analysis of the genotypes rather than the combined alleles, as well as using multiple measures of episodic memory performance. The results showed no influence of *KIBRA* genotypes on visual and verbal episodic memory performance in healthy older adults.

## Conflict of interest statement

Dr. M. Summers reports personal fees from Eli Lilly (Australia) Pty Ltd, grants from Novotech Pty Ltd, outside the submitted work.

## References

[B1] Almeida O. P. Schwab S. G. Lautenschlager N. T. Morar B. Greenop K. R. Flicker L. (2008). KIBRA genetic polymorphism influences episodic memory in later life, but does not increase the risk of mild cognitive impairment. J. Cell. Mol. Med. 12, 1672–1676 10.1111/j.1582-4934.2008.00229.x 18194457 PMC3918083

[B2] Bates T. C. Price J. F. Harris S. E. Marioni R. E. Fowkes F. G. Stewart M. C. (2009). Association of KIBRA and memory. Neurosci. Lett. 458, 140–143 10.1016/j.neulet.2009.04.050 19397951

[B3] Burgess J. D. Pedraza O. Graff-Radford N. R. Hirpa M. Zou F. Miles R. (2011). Association of common KIBRA variants with episodic memory and AD risk. Neurobiol. Aging 32, 557.e1–557.e9 10.1016/j.neurobiolaging.2010.11.004 21185624 PMC3065956

[B4] Cambridge Cognition (2004). Cambridge Automated Neuropsychological Test Assessment Battery. Cambridge: Cambridge Cognition Ltd

[B5] Corder E. H. Saunders A. M. Strittmatter W. J. Schmechel D. E. Gaskell P. C. Small G. W. (1993). Gene dose of apolipoprotein E type 4 allele and the risk of Alzheimer’s disease in late onset families. Science 261, 921–923 10.1126/science.8346443 8346443

[B6] Cosentino S. Scarmeas N. Helzner E. Glymour M. M. Brandt J. Albert M. (2008). APOE epsilon 4 allele predicts faster cognitive decline in mild Alzheimer disease. Neurology 70, 1842–1849 10.1212/01.wnl.0000304038.37421.cc 18401023 PMC2676693

[B7] Dickerson B. C. Eichenbaum H. (2010). The episodic memory system: neurocircuitry and disorders. Neuropsychopharmacology 35, 86–104 10.1038/npp.2009.126 19776728 PMC2882963

[B8] Hariri A. R. Goldberg T. E. Mattay V. S. Kolachana B. S. Callicott J. H. Egan M. F. (2003). Brain-derived neurotrophic factor Val66Met polymorphism affects human memory-related hippocampal activity and predicts memory performance. J. Neurosci. 23, 6690–6694 0270-6474/03/236690-05 12890761 10.1523/JNEUROSCI.23-17-06690.2003 PMC6740735

[B9] Kathiresan S. Newton-Cheh C. Gerszten R. E. (2004). On the interpretation of genetic association studies. Eur. Heart J. 25, 1378–1381 10.1016/j.ehj.2004.06.035 15321696

[B10] Kauppi K. Nilsson L. G. Adolfsson R. Eriksson E. Nyberg L. (2011). KIBRA polymorphism is related to enhanced memory and elevated hippocampal processing. J. Neurosci. 31, 14218–14222 10.1523/JNEUROSCI.3292-11.2011 21976506 PMC6623644

[B11] Kremerskothen J. Plaas C. Büther K. Finger I. Veltel S. Matanis T. (2003). Charatierization of KIBRA, a novel WW domain-containing protein. Biochem. Biophys. Res. Commun. 300, 862–867 10.1016/s0006-291x(02)02945-5 12559952

[B12] Laukka E. J. Lövdén M. Herlitz A. Karlsson S. Ferencz B. Pantzar A. (2013). Genetic effetcs on old-age cognitive functioning: a population-based study. Psychol. Aging 28, 262–274 10.1037/a0030829 23276211

[B13] Lynch M. A. (2004). Long-term potentiation and memory. Physiol. Rev. 84, 87–136 10.1152/physrev.00014.2003 14715912

[B14] Makuch L. Volk L. Anggono V. Johnson R. C. Yu Y. Duning K. (2011). Regulation of AMPA receptor function by the human memory-associated gene KIBRA. Neuron 71, 1022–1029 10.1016/j.neuron.2011.08.017 21943600 PMC3200575

[B15] McClearn G. E. Johansson B. Berg S. Pedersen N. L. Ahern F. Petrill S. A. (1997). Substantial genetic influence on cognitive abilities in twins 80 or more years old. Science 276, 1560–1563 10.1126/science.276.5318.1560 9171059

[B16] Milnik A. Heck A. Vogler C. Heinze H. J. de Quervain D. J. F. Papassotiropoulos A. (2012). Association of *KIBRA* with episodic and working memory: a meta-analysis. Am. J. Med. Genet. B Neuropsychiatr. Genet. 159, 958–969 10.1002/ajmg.b.32101 23065961

[B17] Mormino E. C. Kluth J. T. Madison C. M. Rabinovici G. D. Baker S. L. Miller B. L. (2009). Episodic memory loss is related to hippocampal-mediated beta-amyloid deposition in elderly subjects. Brain 132, 1310–1323 10.1093/brain/awn320 19042931 PMC2677792

[B18] Nacmias B. Bessi V. Bagnoli S. Tedde A. Cellini E. Piccini C. (2008). KIBRA gene variants are associated with episodic memory performance in subjective memory complaints. Neurosci. Lett. 436, 145–147 10.1016/j.neulet.2008.03.008 18378080

[B19] Need A. C. Attix D. K. McEvoy J. M. Cirulli E. T. Linney K. N. Wagoner A. P. (2008). Failure to replicate effect of Kibra on human memory in two large cohorts of European origin. Am. J. Med. Genet. B Neuropsychiatr. Genet. 147, 667–668 10.1002/ajmg.b.30658 18205171

[B20] Papassotiropoulos A. Stephan D. A. Huentelman M. J. Hoerndli F. J. Craig D. W. Pearson J. V. (2006). Common Kibra alleles are associated with human memory performance. Science 314, 475–478 10.1126/science.1129837 17053149

[B21] Preuschhof C. Heekeren H. R. Li S. C. Sander T. Lindenberger U. Bäckman L. (2010). KIBRA and CLSTN2 polymorphisms exert interactive effects on human episodic memory. Neuropsychologia 48, 402–408 10.1016/j.neuropsychologia.2009.09.031 19804789

[B22] Schaper K. Kolsch H. Popp J. Wagner M. Jessen F. (2008). KIBRA gene variants are associated with episodic memory in healthy elderly. Neurobiol. Aging 29, 1123–1125 10.1016/j.neurobiolaging.2007.02.001 17353070

[B23] Schneider A. Huentelman M. J. Kremerskothen J. Duning K. Spoelgen R. Nikolich K. (2010). KIBRA: a new gateway to learning and memory? Front. Aging Neurosci. 2:4 10.3389/neuro.24.004.2010 20552044 PMC2874402

[B24] Sédille-Mostafaie N. Sebesta C. Huber K. R. Zehetmayer S. Jungwirth S. Tragl K. H. (2012). The role of memory-related gene polymorphisms, KIBRA and CLSTN2, on replicate memory assessment in the elderly. J. Neural Transm. 119, 77–80 10.1007/s00702-011-0667-9 21643791

[B25] Slooter A. J. Cruts M. Kalmijn S. Hofman A. Breteler M. M. Van Broeckhoven C. (1998). Risk estimates of dementia by apolipoprotein E genotypes from a population-based incidence study: the Rotterdam study. Arch. Neurol. 55, 964–968 10.1001/archneur.55.7.964 9678314

[B26] Squire L. R. (2004). Memory systems of the brain: a brief history and current perspective. Neurobiol. Learn. Mem. 82, 171–177 10.1016/j.nlm.2004.06.005 15464402

[B27] Strauss E. Sherman E. M. S. Spreen O. (2006). A Compendium of Neuropsychological Tests: Administration, Norms and Commentary 3rd Edn. New York, NY: Oxford University Press

[B28] Summers M. J. Saunders N. L. J. Valenzuela M. J. Summers J. J. Ritchie K. Robinson A. (2013). The Tasmanian Healthy Brain Project (THBP): a prospective longitudinal examination of the effect of university-level education in older adults in preventing age-related cognitive decline and reducing the risk of dementia. Int. Psychogeriatr. 25, 1145–1155 10.1017/s1041610213000380 23522602

[B29] Vassos E. Bramon E. Picchioni M. Walshe M. Filbey F. M. Kravariti E. (2010). Evidence of association of KIBRA genotype with episodic memory in families of psychotic patients and controls. J. Psychiatr. Res. 44, 795–798 10.1016/j.jpsychires.2010.01.012 20185150

[B30] Wang D. Liu B. Qin W. Wang J. Zhang Y. Jiang T. (2013). KIBRA gene variants are associated with synchronization within the default-mode and executive control networks. Neuroimage 69, 213–222 10.1016/j.neuroimage.2012.12.022 23266749

[B31] Wersching H. Guske K. Hasenkamp S. Hagedorn C. Schiwek S. Jansen S. (2011). Impact of common KIBRA allele on human cognitive functions. Neuropsychopharmacology 36, 1296–1304 10.1038/npp.2011.16 21346737 PMC3079841

[B32] Yasuda Y. Hashimoto R. Ohi K. Fukumoto M. Takamura H. Iike N. (2010). Association study of KIBRA gene with memory performance in a Japanese population. World J. Biol. Psychiatry 11, 852–857 10.3109/15622971003797258 20509760

[B33] Zakzanis K. K. (2001). Statistics to tell the truth, the whole truth and nothing but the truth: formulae, illustrative numerical examples and heuristic interpretation of effect size analyses for neuropsychological researchers. Arch. Clin. Neuropsychol. 16, 653–667 10.1093/arclin/16.7.653 14589784

